# A Case of Recurrent Thyrotoxicosis in a Thyroglossal Duct Cyst 18 Years Following Thyroid Surgery

**DOI:** 10.7759/cureus.39829

**Published:** 2023-06-01

**Authors:** Mariam H Baghaffar, Shaza Samargandy

**Affiliations:** 1 Internal Medicine, King Abdulaziz University Faculty of Medicine, Jeddah, SAU; 2 Endocrinology, King Abdulaziz University Faculty of Medicine, Jeddah, SAU

**Keywords:** sub-total thyroidectomy, thyroglossal duct cyst, thyrotoxicosis, recurrence, graves’ disease

## Abstract

Graves' disease (GD) is an immune-mediated condition related to high thyroid-stimulating immunoglobulin levels. Here, we present a rare case of recurrent thyrotoxicosis that developed in a thyroglossal duct cyst (TGDC) and the residual thyroid tissue in a 46-year-old female following subtotal thyroidectomy. In 2005, she was diagnosed with GD causing thyrotoxicosis and treated with subtotal thyroidectomy. In 2022, she was seen at our clinic with a neck swelling gradually growing in size over the last 10 years. On examination, the mass was found to be moving with tongue protrusion. She was on thyroxin 100 mcg daily, and the dose was reduced gradually until she was maintained on no therapy for hypothyroidism and was still thyrotoxic. The combined clinical, laboratory thyroid scintigraphy and ultrasonographic features favored early developing recurrent Graves' disease in the thyroid residual and TGDC. She was started on carbimazole and was referred for surgery. Our case represents a rare occurrence of recurrent GD in the thyroid residual and TGDC.

## Introduction

Graves' disease (GD), which is an immune-mediated condition, is characterized by high thyroid-stimulating immunoglobulin levels [1]. The prevalence of thyroid disease widely varies by age, sex, geographic area, and ethnicity [2-5]. Between the ages of 20 and 50, it is the most frequent cause of hyperthyroidism, accounting for 60%-80% of all cases and is more common in females [2]. The reported prevalence is 0.8% in the USA, 1.3% in Europe, and 2.8% in Saudi Arabia [3,4]. It occurs with the same frequency in Caucasians, Hispanics, and Asians but at lower rates in African Americans [5].

The treatment of Grave’s disease includes antithyroid drugs such as methimazole and propylthiouracil, radioactive iodine therapy (RAI), and thyroidectomy [6]. Regarding the surgical method, there has been a tendency for total or near total thyroidectomy in treating GD as it produces more satisfactory outcomes than a subtotal thyroidectomy due to a reduced rate of recurrent thyrotoxicosis [7]. The thyroid gland descends from the foramen cecum to its final position during gestation, creating the thyroglossal duct tract. Although the tract typically atrophies, some patients may still have thyroid tissue and tract remnants [8]. Here, we present a rare case of recurrent thyrotoxicosis in a newly developed thyroglossal duct cyst 18 years post-subtotal thyroidectomy.

## Case presentation

**Figure 1:**
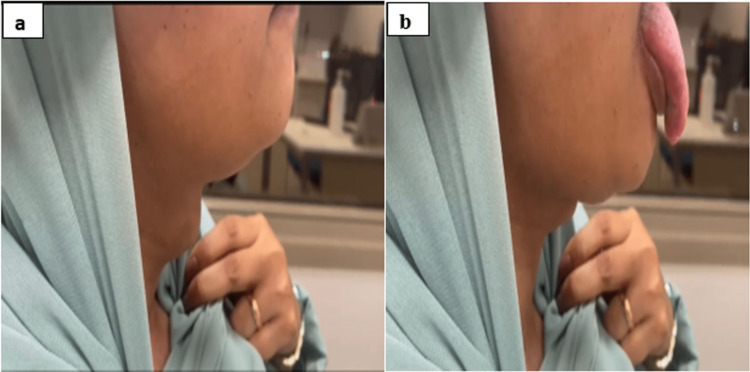
Images showing (a) a non-tender midline neck swelling (4 x 3 cm) and (b) the swelling moving slightly with tongue protrusion

A 46-year-old woman was diagnosed with GD thyrotoxicosis and had subtotal thyroidectomy surgery in 2005 as a mode of treatment. Based on her preference, the patient did not provide any medical reports or details from her initial presentation or surgery. She did mention that she was later maintained on thyroxin 100 mcg daily. In 2012, she noticed a midline neck swelling that grew slowly but remained stable at the current size in the last 10 years. Subsequently, she sought medical advice at the endocrine clinic for further evaluation. She also mentioned that her primary care physician had reduced her thyroxin doses over the past year due to abnormal laboratory test results, and lately, she had been only on 25 mcg daily. She also gave a history of upper respiratory tract infections (URTI) every six months. By physical examination, the midline neck swelling, measuring approximately 4 x 3 cm, was non-tender and was moving with tongue protrusion (Figure 1). No palpable lymph nodes were detected. She was clinically diagnosed with a thyroglossal cyst.

**Figure 2:**
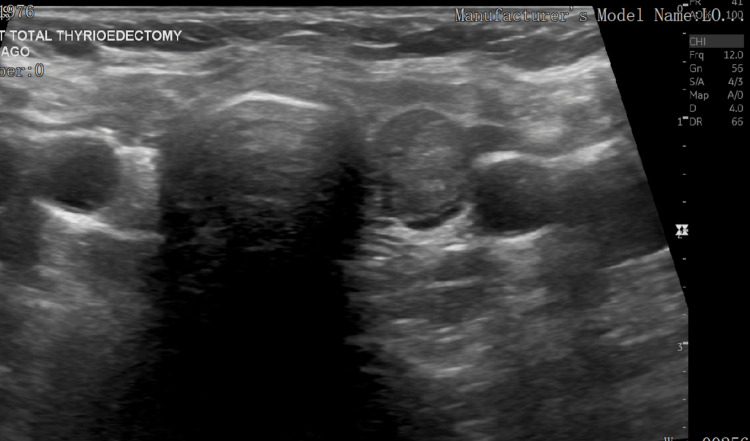
A neck ultrasound scan showing a midline exophytic thyroid nodule measuring about 2.8 x 16 cm

The thyroid function test (TFT) reported a suppressed thyroid stimulating hormone (TSH) level of <0.15 mIU/L (normal, 0.5-5.0 mIU/L) and normal free thyroxine (FT4), while the tri-iodothyronine (T3) level was not tested. The neck ultrasound scan showed a midline exophytic thyroid nodule measuring about 2.8 x 16 cm, with a heterogeneous echo pattern and increased vascularity by color Doppler ultrasound (Figure 2). Another nodule was seen in the operative bed of the left thyroid lobe measuring 1.2 x 1 cm. No retrosternal extension was observed. Bilateral enlarged reactive cervical lymphadenopathy was observed. The final opinion was midline mass suggestive of a thyroglossal duct cyst and a left-sided thyroid bed nodule.

**Figure 3:**
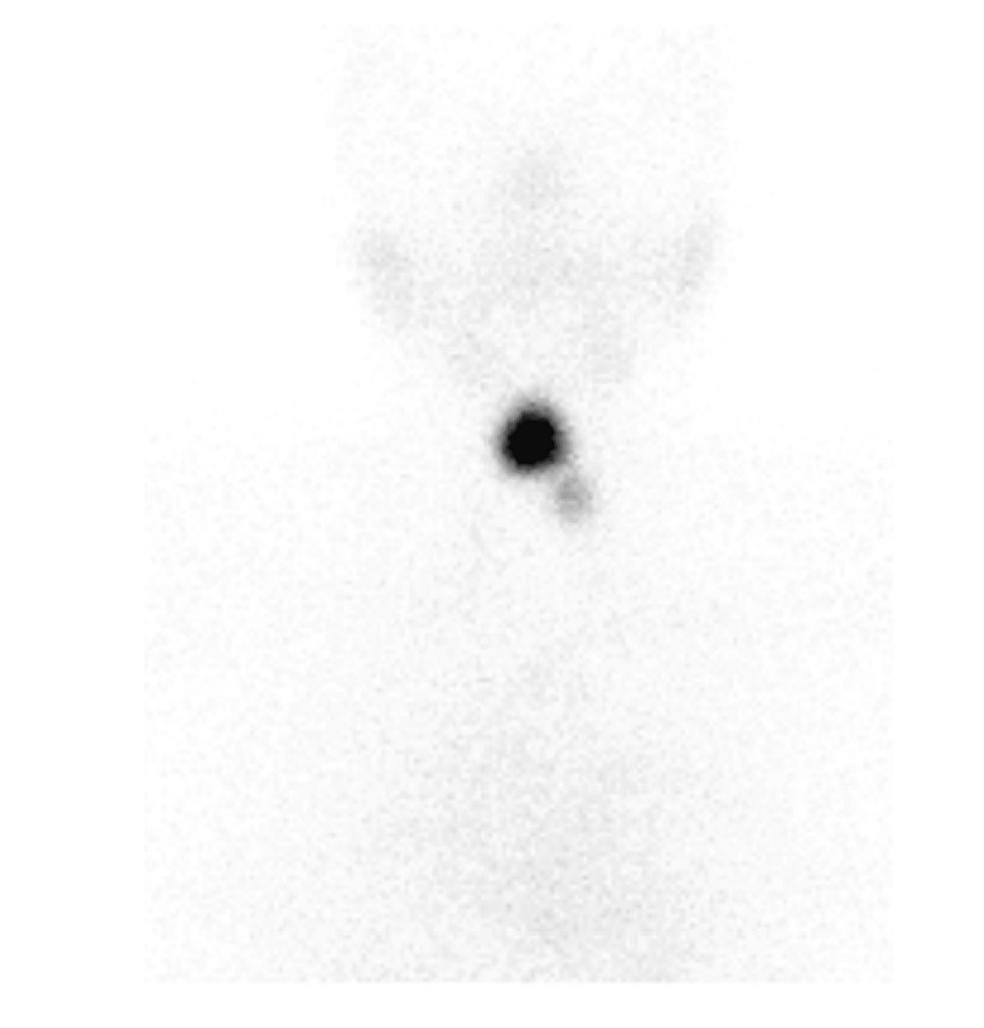
Thyroid scintigraphy with the uptake demonstrating an intense tracer uptake in the midline

Levothyroxine was discontinued, and the patient was re-evaluated six weeks later with a TFT that showed TSH 0.018 mIU/L, FT3 1.46 pmol/L (normal, 2.0-7.0 pmol/L), FT4 1.33 ng/dL (normal, 0.7-1.9 ng/dL), and a TSH receptor antibodies level of 2.8 IU/L (<2.9, negative results; >3.3, positive results). Nuclear medicine (NM)-thyroid scintigraphy with uptake demonstrated an intense tracer uptake in the midline, exhibiting a relatively uniform tracer uptake pattern with no sizable cold nodules detected (Figure 3). Another smaller area of a faint tracer uptake was seen in the presumed site of the left lobe. No retrosternal activity was seen. The extra-thyroidal background activity and salivary glands were partially depleted from their ordinary share in tracer handling. The thyroid uptake percentage was 3.7% (normal, 0.5%-4%), which showed a high normal thyroid uptake value. Scintigraphic features were suggestive of thyroid tissue in the midline of the neck as well as a small residual left thyroid lobe.

The combined scintigraphy features (a high normal thyroid uptake) and laboratory data favored early developing recurrent GD. She was started on carbimazole and was referred to an ear, nose, and throat (ENT) surgeon for surgery.

## Discussion

Hyperthyroidism caused by Graves' disease is treated by partial thyroidectomy, by decreasing the gland's ability to produce thyroxine [9]. Nevertheless, even following a radical, total thyroidectomy, 10% of GD patients develop recurrent thyrotoxicosis [10]. Following a subtotal thyroidectomy, thyrotoxicosis may return due to a latent, persistent disease [11]. Additionally, ectopic and aberrant thyroid tissue sites may develop recurrent GD [12]. Ectopic thyroid tissue develops along the midline or just lateral to the midline along the thyroglossal duct as a result of abnormal thyroid gland descent during gestation. Since ectopic thyroid tissue is frequently asymptomatic, its exact incidence is not clearly identified. Thyroglossal duct cysts, which are expansions of the thyroglossal tract and may be lined with thyroid tissue, can be confused with ectopic thyroid tissue, causing the recurrence of GD [13]. According to a recently released treatment guideline from the American Thyroid Association and the American Association of Clinical Endocrinologists, these sites may include the anterior neck, thyroglossal duct remnants, mediastinum, and lateral neck [12]. Surgery is chosen as the primary treatment for GD; the guidelines recommend near-total or total thyroidectomy due to the high recurrence rates of thyrotoxicosis (10%) following subtotal thyroidectomy [10,12]. Here, we presented a rare case of recurrent thyrotoxicosis following subtotal thyroidectomy, originating from the TGDC in the midline of the neck and a small residual left thyroid lobe. The subtotal thyroidectomy was chosen because the patient did not want to take levothyroxine for the rest of her life.

Scintigraphic features of our case suggested thyroid tissue in the midline of the neck and a small residual left thyroid lobe. The combined scintigraphy features (a high normal thyroid uptake) and clinical and laboratory data favored early developing recurrent Graves’ disease in the thyroid tissue residual and the TGDC. After the diagnosis of our case (a recurrent GD), she was recommended carbimazole and was referred to an ENT surgeon for surgery.

We compared our case to previously published studies evaluating the recurrence of Graves' disease after subtotal thyroidectomy. Based on studies from the early 1970s, the recurrence of thyrotoxicosis following subtotal thyroidectomies is well documented [9]. A study of patients with recurrent thyrotoxicosis after subtotal thyroidectomy has shown that the operation has a profound effect on the natural history of GD. It is followed by pronounced changes in the immunological features of the disease, with a fall in the prevalence of serum thyroid autoantibodies, including the long-acting thyroid stimulator. Thyroid suppression returns to normal in 70% of patients. The treatment produces two populations of patients. In the larger group, there is a permanent remission of the disease process. In the smaller group, the disease process persists and, consequently, recurrent hyperthyroidism may develop. The mechanism of the change in the larger group of patients probably has an immunological basis [9].

Gaschen et al. described a rare case of recurrence of GD in a 27-year-old female who underwent a near-total thyroidectomy and started taking levothyroxine soon after [14]. Off levothyroxine, recurrent thyrotoxicosis became apparent about two years later. An ultrasound scan revealed vascularized thyroid tissue, and a radioactive iodine scan revealed an enhanced uptake in the right thyroid area. She started taking the antithyroid medication, and after reaching the euthyroid state, she received radioactive iodine treatment. Few cases of GD caused by hyperfunction of thyroglossal duct remnants have been reported, and to our knowledge, there have been only three cases of recurrence following total thyroidectomy [15-17]. However, we did not find a similar case after subtotal thyroidectomy. Basili et al. reported of a 40-year-old woman with a history of total thyroidectomy for Graves' disease, who had clinical signs of hyperthyroidism and a midline neck mass that was slowly growing [15]. Thyroid-stimulating hormone was repressed, and serum-free triiodothyronine and thyroxine levels were increased. An infrahyoid-localized solid mass was detected using neck ultrasonography, and an enhanced uptake was also seen at the same level by radionuclide scanning. A 4-cm solid mass was removed using the Sistrunk method. The Sistrunk procedure, which consists of the excision of the thyroglossal duct cyst, the middle part of the hyoid bone, and the surrounding tissue around the thyroglossal tract, is a frequently chosen option for the surgery of a thyroglossal duct cyst, especially for those who are categorized as low-risk patients [16]. According to Vercher-Conejero et al., a 40-year-old woman with GD identified 15 years earlier needed a complete thyroidectomy due to poor disease control. She developed hypothyroidism after surgery, which was treated with l-thyroxine at varied doses based on thyroid function, up to a daily maximum of 175 micrograms [17]. Recently, Vaz-Pereira et al. documented a rare instance of a patient who had undergone a Sistrunk operation and had a recurrence of GD in a thyroglossal duct following total thyroidectomy [18]. To rule out persisting and functional thyroid tissue, patients with a history of GD and impaired thyroid function regulation following complete thyroidectomy should be further examined/evaluated for recurrence of GD. Surgery is a viable choice in these situations.

## Conclusions

Our case is rare for recurrent thyrotoxicosis (GD) following subtotal thyroidectomy and the later manifestation of TGDC. GD recurrence is a failure of surgical treatment. This may depend on patient preference as not all patients would want to take levothyroxine for the rest of their lives as is the case in total thyroidectomies; it may also depend on the severity of the disease, so it's on a case-by-case basis. Patients may opt for a subtotal, while the recurrence is reported to vary.
